# New insights in pediatrics in 2021: choices in allergy and immunology, critical care, endocrinology, gastroenterology, genetics, haematology, infectious diseases, neonatology, neurology, nutrition, palliative care, respiratory tract illnesses and telemedicine

**DOI:** 10.1186/s13052-022-01374-8

**Published:** 2022-11-26

**Authors:** Carlo Caffarelli, Francesca Santamaria, Ettore Piro, Simona Basilicata, Valeria Delle Cave, Marilena Cipullo, Sergio Bernasconi, Giovanni Corsello

**Affiliations:** 1 Department of Medicine and Surgery, Clinica Pediatrica, Azienda Ospedaliera-Universitaria, University of Parma, Via Gramsci 14, Parma, Italy; 2 Department of Translational Medical Sciences, Federico II University, Naples, Italy; 3 Department of Sciences for Health Promotion and Mother and Child Care G. D’Alessandro, University of Palermo, Palermo, Italy; 4 Microbiome Research Hub, University of Parma, Parma, Italy

**Keywords:** Allergy, Immunology, Critical care, Endocrinology, Gastroenterology, Genetics, Hematology, Infectious diseases, Neonatology, Neurology, Nutrition, Palliative care, Respiratory tract illnesses, Telemedicine

## Abstract

In this review, we report the developments across pediatric subspecialties that have been published in the Italian Journal of Pediatrics in 2021. We highlight advances in allergy and immunology, critical care, endocrinology, gastroenterology, genetics, hematology, infectious diseases, neonatology, neurology, nutrition, palliative care, respiratory tract illnesses and telemedicine.

## Introduction

Amid the persistence of the pandemic, the scientific community pushed ahead innovative studies on the conditions apart from Covid-19, most managed by pediatricians. So, in this review, we have looked back on the 2021 most accessed and innovative papers from the Italian Journal of Pediatrics. They offer remarkable advancements in allergy and immunology, critical care, endocrinology, gastroenterology, genetics, hematology, infectious diseases, neonatology, neurology, nutrition, palliative care, respiratory tract illnesses, and telemedicine to improve care of children.

### Allergy and immunology. 1- House dust mite immunotherapy; 2- Eosinophilic esophagitis; 3- Kawasaki disease

#### House dust mite immunotherapy

The heterogeneity of asthma has long been recognized. The allergic asthma is often associated with atopic diseases [1] with eosinophil inflammation [2, 3] acidification [4] and oxidative stress [5] in the airways. Allergen immunotherapy, both subcutaneous immunotherapy and sublingual immunotherapy, remains the only effective treatment for IgE-mediated allergic rhinoconjunctivitis and asthma [6]. It has also been used in skin diseases [7]. The subcutaneous route is recommended in children > 5 years of age because less adherence and lack of quick reporting symptoms of adverse reactions. However, early allergen immunotherapy may have better efficacy. Yang et al. [8] studied safety of house dust mite subcutaneous immunotherapy in children aged 3 to 5 years, with respiratory allergies. The rate of systemic reactions was low. In addition, they were mild, and no anaphylaxis occurred. These findings suggest that subcutaneous immunotherapy appears safe in preschool children.

#### Eosinophilic esophagitis

Both IgE and non-IgE mediated food allergy are frequent [9]. The definitive mean for diagnosing food allergy is the oral food challenge [10], that is risky [11] and time consuming. Predictors of reactions included specific IgE levels and skin tests to the food [12, 13]. Other biomarkers such as intestinal permeability has been proposed [14]. In eosinophilic esophagitis, Barni et al. [15] reported that proton pump inhibitors, corticosteroids and elimination diets are the first option for treatment. However, the identification of causal foods to be eliminated from the diet presents a diagnostic challenge. Elimination diet can be based on allergy testing results. Another possibility is an empiric restrict diet that eliminate the most frequent foods causing allergic reactions, for example the six-food elimination diet (cow’s milk, egg, soy, wheat, peanuts/tree nuts, and fish/shellfish). An elemental diet without allergenic proteins is the most successful diet and it is also more effective than other first option of treatment. The choice of treatment should be taken after considering preferences of parents. Biologics such as dupilumab seems to be a promising future option for treating eosinophilic esophagitis.

#### Kawasaki disease

Italian guideline on Kawasaki Disease has been updated last year [16]. It recommends against waiting more than 10 days before starting treatment with intravenous immunoglobulin plus medium–high dosage of acetylsalicilic acid that has an antinfiammatory effect. Children at high risk are better defined. They include: < 12 month of age, PCR > 200 mg/L at onset, ipertransaminasemia, albumin < 2.5 g/dL), severe anemia at onset, coronary artery aneurysms, macrophage activation syndrome or septic shock. High risk children should be treated with intravenous immunoglobulin (plus low dose acetylsalicilic acid) and IV methylprednisolone (30 mg/kg/day). Among medication to achieve control, evidence on the efficacy of infliximab [17], anakinra [18] and canakinumab in children with resistant Kawasaki Disease have been provided.

### Critical care. 1- Cardiopulmonary resuscitation; 2- Psychosomatic pain

#### Cardiopulmonary resuscitation

There is a long-standing debate on the survival following discharge from hospital in pediatric patients who performed cardiopulmonary resuscitation for in-hospital cardiac arrest with cardiac activity cessation [19]. Bimerew et al. [20] undertook a systematic review and meta-analysis on this uncommon event [21]. They included 25 studies with 28,479 patients. The prevalence of survival was 48%. Living in Asiatic countries and in low- or middle-income countries was associated with decreased survival. Caution on the evidence provided by the study, is justified by high heterogeneity on the reported prevalence of survival and by lack of considering factors such as chest compression depth and rate that were found to be associated with survival outcomes [22]. Nonetheless, the study showed that most pediatric patients with in-hospital cardiopulmonary resuscitation procedures failed to survive after hospital discharge. The Authors suggest that new strategies are warranted for cardiopulmonary resuscitation in children.

#### Psychosomatic pain

Several papers have recently elucidated the clinical care related to psychosomatic pain [23]. The PIPER (Pain in Paediatric Emergency Room) group comprises pediatricians, emergency physicians, and anesthesiologists from 52 Italian emergency departments [24]. It presented an evidence-based approach on clinical features and management of patients with psychosomatic pain in the pediatric emergency department. The paper highlighted differences in clinical manifestations between patients. Psychosomatic pain is often localized at head or abdomen. It can also be simultaneously present in different sites or be migrant. It is often every day associated with marked fatigue, limiting daily activities. It persists for months or years. The treatment still relies upon psychological assistance for both patients and families [25]. An effective analgesic is lacking. Pharmacological treatment should be used only for psychiatric comorbidities. The Authors conclude that standardization of care in the emergency department is warranted.

### Endocrinology. 1- Faltering growth; 2- Phelan-McDermid syndrome

#### Faltering growth

The meaning of the term Failure to thrive is discussed. Numerous definitions have been given, generally based on the evaluation of anthropometric parameters (in particular on a significant decrease in the growth rate) which do not agree with each other and therefore there does not seem to be a specific parameter to identify patients to be included in this diagnostic category. It is also hypothesized that failure to thrive cannot constitute a true category but that it is a clinical signal of an insufficient nutritional intake to guarantee growth. The term “faltering growth” has recently begun to be proposed to replace failure to thrive [1]. The etiology of faltering growth is very heterogeneous and traditionally two categories of causes are distinguished: organic and non-organic, although sometimes this distinction is considered obsolete because there may be an interplay of organic, behavioural, nutritional and psychosocial factors [26]. However, there is a trend to deepen the multifactorial links between faltering growth and malnutrition considered as a signal of imbalance between nutritional intakes and macro and micronutrient requirements [27]. Generally, the presence of an organic cause is suspected on the basis of a detailed medical history and the presence of specific signs and symptoms and confirmed by specific tests. It is interesting to note that in large series of hospitalized patients diagnosed with failure to thrive in which patients with recognized organic disease were excluded the most common final diagnosis was “purely nutrition” failure to thrive because of inadequate caloric intake, and that extensive diagnostic workup was rarely helpful to reveal the etiology [28] In conclusion: 1) an important diagnostic role is played by the family pediatrician who can, with a few targeted tests, make an initial diagnosis; 2) hospitalization should be reserved for patients with specific characteristics and it is useful for the diagnostic procedure to follow specific flow charts step by step to avoid unnecessary laboratory and / or radiological investigations [29]; 3) in most cases the therapy involves the collaboration of a team that can intervene on the nutritional and behavioral aspects.

#### Phelan-McDermid syndrome

Phelan-McDermid syndrome (is a rare genetic syndrome originally considered to be the expression of a 22q13 deletion and more specifically of alterations in the SHANK3 gene. More recently it has been proposed to include into this classification also patients with interstitial deletions close to the locus of this gene and not involving SHANK3 [30]. In fact, these patients present a clinical picture largely overlapping with that of SHANK3 involvement [31, 32]. The syndrome is characterized by intellectual disability, hypotonia, delayed or absent speech, motor impairment, behavioral anomalies, and dysmorphic features [31]. Moreover, autism spectrum disorders may be present in over 75% of patients. Various pharmacological substances (intranasal insulin, lithium) have been used in clinical trials and among these we must mention the insulin-like growth factor-1. Based on the results obtained with the latter, Xie et al. [32] verified the efficacy of a treatment with recombinant human growth hormone in one patient demonstrating that with this hormone it is possible to obtain results similar to those obtained with the insulin-like growth factor in the absence of significant side effects. In particular, an improvement in motor skills and autism-like behaviors is reported. This positive effect was subsequently confirmed in an open-label, cross-over study on a small series of patients [33]. Furthermore, these results are in line with experimental models in the rat that have shown that insulin-like growth factor-1 may increase synaptic plasticity and improve both motor and behavioral deficits characteristic of Phelan-McDermid syndrome. Even if the results are not definitive, a new therapeutic perspective is opening which could be based on single pharmacological products or on the joint or serial use of some of them.

### Gastroenterology. 1-Oral ulcers; 2- Haemolytic uremic syndrome; 3- Inflammatory bowel disease and glycogen storage disease

#### Oral ulcers

Aphthous stomatitis is a common disease in childhood [34]. Legeret et al. [35] have performed a systematic review to explore causes and treatment of oral ulcers. The pathogenesis has been found to be multifactorial and they can be elicited by infections, autoimmune diseases, drugs, immunosuppression, gastrointestinal diseases, nutritional deficiency or local trauma. There is controversy about the optimal treatment. Avoidance and prevention of exposure to triggering agents are necessary as well as supplementation of vitamins and nutrients when needed. Pain can be relieved by local anesthetics such as lidocaine and benzocaine, especially at meals. Chlorhexidine and topical antibiotics can be effective in controlling infections, especially secondary [36]. Topical corticosteroids are a useful first-line option for reducing ulcers [36]. When topical treatment is not effective, a course of oral corticosteroids may be of benefit [37]. Montelukast, colchicine, dapsone and thalidomide have been also proposed as second-line options.

#### Haemolytic uremic syndrome

Hemolytic uremic syndrome (HUS) is a rare disorder caused by strains of Escherichia coli (E. coli) releasing Shiga-like toxin in up to 90% of cases. The condition is characterized by microangiopathic hemolytic anemia, thrombocytopenia, and acute kidney injury. Central nervous system involvement is the most common extra-renal disorder in typical hemolytic uremic syndrome. Major symptoms such as seizures, impaired consciousness, and stroke were seen in 33% of cases while minor symptoms in 14% [38]. Eculizumab, an anti-C5-convertase monoclonal antibody, seems to be helpful for treating children with central nervous system symptoms [39]. Peripheral nerve involvement is very rare. Santangelo et al. [40] showed patients with typical hemolytic uremic syndrome with late onset inflammatory polyneuropathy after acute treatment with eculizumab and renal replacement therapy and resolution of central nervous system (injury. These children received methylprednisolone pulses (and immunoglobulins or plasma exchange, followed by a prolonged multidisciplinary neurorehabilitation program. A full functional improvement occurred after intensive 8 months of rehabilitation. The delayed occurrence of peripheral symptoms [40] might indicate that lack of nutrients (i.e., probiotics or vitamins) [41] may lead to peripheral neuropathy [42]. Authors concluded that neuro-rehabilitation may significantly improve the outcome of children with polyneuropathy during hemolytic uremic syndrome.

#### Inflammatory bowel disease and glycogen storage disease

Glycogen storage disease type Ib is an autosomal recessive disorder due to microsomal glucose-6-phosphate transporter deficiency. Glycogen storage disease type Ib patients [43] present hypoglycemia, glycogen accumulation in liver and kidney, neutropenia, neutrophil dysfunction, and increased risk of inflammatory bowel disease (i.e., Crohn disease-like enterocolitis). Treatment is based on frequent intake of uncooked cornstarch and granulocyte-colony stimulant factor for improving neutropenia and bacterial infection. Granulocyte-colony stimulant factor is associated with severe side effects such as acute myeloid leukemia, myelodysplasia. Granulocyte-colony stimulant factor is useful on IBD in some patients with glycogen storage disease type Ib [44]. Empagliflozin is useful in type 2 diabetes. It reduces renal 1,5-anhydroglucitol resorption leading to decrease accumulation in neutrophils that impairs survival and function in glycogen storage disease type Ib [45]. In glycogen storage disease type Ib and severe IBD, Rossi et al. [46] has found that empagliflozin improved gastrointestinal symptoms in a week and granulocyte-colony stimulant factor dose was reduced by one third. Abdominal magnetic resonance imaging showed amelioration of disease activity after 3 months. Authors concluded that empagliflozin was effective with no side effects.

### Genetics. 1- White-Sutton syndrome; 2- Aromatic L-amino acid decarboxylase deficiency; 3- 15q26 deletion

#### White-Sutton syndrome

The report on White-Sutton syndrome [47] is part of the interesting line of research on genetic causes underlying developmental anomalies [48]. Specifically, the clinical contribution expanding the neurological and behavioral phenotype refers to a patient with White-Sutton syndrome, related to a novel heterozygous pogo transposable element with znf domain (POGZ) mutation [49]. The patient showed a moderate intellectual impairment, mild autistic traits and the never described co-existence of both paroxysmal not-epileptic events and abnormal EEG without clinical seizures. An important aspect of the article is, for those involved in clinical research on genetic syndromes associated with neurodevelopmental involvement, the wide and updated arsenal of specific diagnostic techniques relating to the different developmental areas that the authors have explored to define the patient's profile.

#### Aromatic L-amino acid decarboxylase deficiency

A modified Delphi consensus method, based on the selection of items drawn from synthesized reviews of the literature rather than open-ended questions, has been adopted as main stage of development of the identification process concerning presentation, diagnosis, and treatment of Aromatic L-amino Acid Decarboxylase deficiency [50]. It is an untreatable rare autosomal recessive neurometabolic disorder, is characterized by a severe impairment of serotonin, dopamine, norepinephrine, and epinephrine biosynthesis causing more often early onset of a severe and progressive neurodevelopmental impairment, although a milder clinical expression has also been described. In relation to the difficulties of early diagnosis the Italian working group and a patients’ association have expressed consensus with high rates of agreement on a series of statements paving the way to disseminate clear messages concerning disease presentation, diagnosis [51] and treatment [52, 53] as well as strategic interventions to disseminate knowledge at different levels.

#### 15q26 deletion

A patient had a complex phenotype characterized by developmental, growth and heart involvement. Array analysis, following a high-resolution R-banded karyogram, identified a 9.15 Mb deletion within the 15q26.1-q26.3 region that was not previously reported [54]. Following the Hugo gene nomenclature committee, the deleted segment encompassed 36 genes, 19 of whom were referenced in the OMIM database, and among which only IGF1R, NR2F2, CHD2 and MEF2A, were consistent with the described patient’s phenotype [55, 56]. Following the authors’ opinion, 15q26 monosomy should be taken in consideration in the differential diagnosis process when growth retardation is associated with congenital heart defect, mainly atrioventricular septal defects and /or aortic arch anomaly [57].

### Hematology. 1- Thalassemia major; 2- Hemoglobin life-threatening levels

#### Thalassemia major

Endocrine complications are very common in multi‑transfused thalassemia major patients. For this reason, international organizations such as the International Network on Endocrine Complications in Thalassemia (I ‑ CET) have published guidelines for the monitoring of endocrine diseases in thalassemia major patients [58]. Most studies on the incidence of endocrinopathies in thalassemia major patients are retrospective and performed on adult patient populations. It may therefore be interesting to evaluate this situation in a pediatric population with the aim both of better understanding the natural history of the various endocrine diseases and of implementing appropriate preventive measures as soon as possible. In the survey carried out by Mahmood et al. on 120 patients the presence of endocrine disorders was observed in 23.33% of them. Subclinical or clinical hypothyroidism, abnormalities in glucose homeostasis and hypoparathyroidism are the three endocrine pathologies found [59]. The abnormalities in glucose homeostasis which in perspective can lead to an overt diabetes mellitus deserve a particular comment, suboptimal iron chelation and presence of hepatitis C infection may be a risk factor for the development of insulin glucose intolerance through the induction of insulin resistance [60]. However, it is interesting that 70% of the patients studied showed signs of malnutrition [59]. This aspect deserves to be investigated because recent research leads to consider that malnutrition at any postnatal age can have both acute and long-term adverse effects on pancreas function [61].

#### Hemoglobin life-threatening levels

Overall, WHO [62] reported anemia in 42% of children under 5 years-old of age. Parodi et al. [63] provided new information concerning good clinical condition with only slowly increasing asthenia despite of unanticipated severe microcytic anemia (hemoglobin 1.9 g/dL, mean corpuscular value 64 fl) and iron deficiency (iron binding capacity 6%, reticulocyte hemoglobin content 15.2 pg) in a girl of African origin aged 5 years. The condition was determined by iron deficiency [64]

and homozygous sickle cell disease [65]. The patient received intravenous packed red blood cells (15 ml/kg) to get 7.3 g/dL hemoglobin level and after a few days, she was discharged with 9.8 g/dL hemoglobin concentration. The findings show that life-threatening hemoglobin values are practically asymptomatic when anemia gradually get worse.

### Infectious diseases. 1-Acute myositis; 2- Hookworm infection; 3- Hematogenous osteomyelitis; 4- Nasal influenza vaccine; 5- Gentamicin; 6- Epstein-Barr virus and hemophagocytic syndrome

#### Acute myositis

Limited studies on cohort of children with benign acute myositis are available. It is overlooked since there is commonly spontaneous resolution and occurrence of rhabdomyolysis, and kidney impairment has been hardly ever described [66]. Brisca et al. [67] identified 113 children diagnosed with benign acute myositis from database of two pediatric Emergency Departments. All patients had normal neurological findings, and no one developed myoglobinuria, or renal failure, accordingly with previous studies [68]. Creatine kinase ranged from 257–12,858 U/L, normal values < 150 U/L. In 51/74 cases (69%) an association with viral infection was found. In agreement with previous findings, influenza viruses were the commonest (37 cases) [68]. Myositis can be a complications of severe acute respiratory syndrome coronavirus-2 and more cases with this virus are expected [69]. Ten percent of children had recurrent episodes. The median serum creatine kinase levels were significantly higher in “recurrent” type than in “non-recurrent” type. Authors concluded that most patients can be treated at home with correct fluid intake, analgesics, and resting period.

#### Hookworm infection

Hookworm infection is common in rural areas of developing countries. In infancy it is rarely reported [70]. Umbrello et al. [71] reported their experience with an Italian 2-month-old infant with vomiting, weight loss, anemia, eosinophilia, and eggs of Ancylostoma duodenale in stools. Treatment with oral mebendazole was successful. Hookworm infection was not reported in the area where infant lived [72]. The mother travelled in an endemic country during pregnancy and had gastrointestinal symptoms. However, parents’ stools resulted negative. So, Authors suggested that vertical transmission of Ancylostoma infection during pregnancy may explain the parasitic disease. Although the response to mebendazole was excellent without side effects [73], the best clinical management needs to be elucidated.

#### Hematogenous osteomyelitis

A multidisciplinary experts reach a consensus by Delphi method on acute hematogenous osteomyelitis [74]. A systematic review retrieved 53 relevant articles that were included in the Consensus. Regarding intravenous antibiotics, the Panel recommended that the first option was ampicillin-sulbactam or cephazolin + gentamycin in infants under 3 months of age; cephazolin in children aged 3 months—5 years to cover K. kingae [75]; oxacillin or cephazolin or clindamycin in children > 5 years. the switch from intravenous to oral therapy is still a matter of debate. Defervescence or decreasing body temperature for 24–48 h, improvement of local symptoms, lack of complication, 30–50% decrease of CRP and pathogens sensible to antibiotic may be considered for switching after 2–4 days of intravenous antibiotics. In infants and children > 3 months of age, the panel recommended to switch to oral therapy within 5–7 days of intravenous antibiotics. Oral treatment with cephalexin or amoxicillin-clavulanic acid, possibly associated with rifampicin should be given. Among the antistaphylococcal penicillin, flucloxacillin, should be the first choice. The length of anticrobial treatment may vary from 3 to 6 weeks, mean 4 weeks [76, 77].

#### Nasal influenza vaccine

The availability of live attenuated influenza vaccine (LAIV) for intranasal administration has given clinicians choices for individual patients. It seems more effective than intramuscular one in children [78, 79]. Gasparini et al. [80] assessed efficacy and parent’s satisfaction of LAIV in 9292 children. They confirmed that rate of adverse events was 24.8%: 80.6% mild, 18.1% moderate and 1.3% significant [81]. Cold (52.5%) and fever (24.4%) were the most frequent. Most parents (83.3%) were very satisfied of LAIV, and they would give further jabs. Most health professional found that LAIV administration was easy. The authors concluded that LAIV is a safe and suitable option.

#### Gentamicin

Gentamicin is a bactericidal aminoglycoside to aerobic Gram-negative bacilli and some Gram-positive cocci with potential risk of nephrotoxicity and ototoxicity [82]. Ghoneim et al. [83] studied MICs of intravenous gentamicin in subjects, aged from 1 month to 12 years of age, using Monte Carlo simulation. They found that a MIC of 2 mg/L is reached using once daily dose of 6–7 mg/kg/day in infants aged 1 month – 12 months and of 4–5 mg/kg/day in children > 1 year of age. These findings extend previous results [84] on effective gentamicin level without toxicity.

#### Epstein-Barr virus and hemophagocytic syndrome

In patients with Epstein-Barr virus (EBV) infection, mononucleosis and hemophagocytic syndrome (HLH), can develop [85]. The most common secondary HLH in children is linked to viral infection, especially EBV [86]. Shi et al. [87] determined the clinical, and laboratory characteristics of the 31 EBV-HLH compared to 61 mononucleosis cases. They noted that cellular immune function was significantly impaired in in the HLH group [88]. Increased D-Dimer level is a predictor of HLH in children with EBV.

### Neonatology. 1- Sepsis; 2- Intravenous sodium bicarbonate; 3- Neonatal seizures; 4- Umbilical venous catheter; 5- Retinopathy of prematurity; 6- Glucose infusion in preterm infants; 7- Respiratory distress syndrome; 8- Delayed cord clamping: 9- Hypercalcemia and hypophosphatemia; 10- Necrotizing enterocolitis; 11- Hypertrophic cardiomyopathy; 12- Asymptomatic hypoglycemia; 13- Congenital hypothyroidism screening

#### Sepsis

Since several studies have showed multiple short and long-term adverse health effects in infants undergoing prolonged antibiotic treatments, reduction of antibiotics administration during the early post-natal period has become a fundamental need. One of the strategies developed to rationalize the use of antibiotics has been the development of tools such as the early onset sepsis risk calculator [89, 90]. In this single-center retrospective study Laccetta et al. [91], assuming an incidence of early-onset sepsis of 2/1000 live births, enrolled a total of 265/1667 (15.9%) newborns ≥ 34 weeks’ gestational age and compared the number of patients for which antibiotics would have been needed, based on early-onset sepsis calculator, and the number of the same patients treated with antibiotics during the study period. The comparison showed in the newborns selected using the early-onset sepsis calculator a lower antibiotic use (12.1%) than the expected (20.7%). The results of this study highlight the need to pursue the best possible antibiotic stewardship, based on a continuous evidence-based update of local guidelines, to further decrease their administration in newborns [92].

#### Intravenous sodium bicarbonate

Intravenous sodium bicarbonate corrects metabolic acidosis, but it is not without potential risks such as fluctuations in cerebral and cardiovascular hemodynamic, increased rates of severe intraventricular hemorrhage and mortality [93]. Massenzi et al. [94] conducted a survey regarding treatment of metabolic acidosis and intravenous sodium bicarbonate prescription habits, involved 117 neonatal intensive care units. The authors used a 14-item questionnaire developed by the Neonatal Pharmacology Study Group of the Italian Society of Neonatology (SIN), that is filled and sent in about 15 min. The study showed that intravenous sodium bicarbonate is a commonly used treatment for metabolic acidosis in more than half of Italian neonatal intensive care units, with significantly variable indications and prescription criteria across centers. They concluded that intravenous sodium bicarbonate should be given to selected cases [95, 96], also considering the severity of intravenous sodium bicarbonate adverse effects and the lack of evidence about its efficacy.

#### Neonatal seizures

The authors of this systematic review deepen the controversies about therapy of neonatal seizures [97]. In recent years, advances in the diagnosis [98] and etiological definition of neonatal seizures have allowed more precise therapeutic approaches [99]. New pharmacological approaches are nowadays available, like sodium channel blockers for genetic early-onset epilepsies as well as the overall good response to levetiracetam of neonatal seizures [100]. Nevertheless, the World Health Organization (WHO) recommends the use of phenobarbital and phenytoin as first-line treatment yet. The authors conclude, that owing to the heterogeneity of the variables considered in the different studies, such as the gestational or postmenstrual age of the samples and the terms of definition of the therapeutic response among the main ones, a persistence of limited available evidence regarding the best pharmacological treatments for neonatal seizures still exist.

#### Umbilical venous catheter

Standard umbilical venous catheter insertion distance is assessed by anthropometric measures or formulas and nomograms usually based on birth weight. Recent guidelines advocate the use of real-time ultrasound to locate umbilical venous catheter tip [101–103]. Rubortone et al. [104] reported on a pre/post interventional study on fifty-four patients to evaluate the efficacy of a training protocol in using ultrasound. A significative improvement in the correct placement of the tip of the catheters was get after the training (75% vs 30.7%, *p* = 0.0023). Interestingly 50% of the positioned catheters underwent migration, as demonstrated by serial scans evaluation included in the protocol. This article offers a valid contribution to the topic and provides useful iconography and detailed description of the protocol used.

#### Retinopathy of prematurity

Dani et al. [105] conducted a study to assess incidence [106] and risk factors [107, 108] for retinopathy of prematurity (ROP) development in preterm infants < 30 weeks gestational age, involving two neonatal intensive care units (NICUs) and 178 infants of which 67 (38%) developed any grade of ROP. Comparison of both the unaffected and affected by ROP sample (stage 1–2-3), using univariate and multivariable logistic regression analyses, allowed to identify maternal milk as protective and intraventricular hemorrhage and RBC transfusion as increasing risk factors for the development of ROP. The occurrence of ROP was similar to that previously reported. Limitations of this study, reported by the authors, are mainly due to the heterogeneity of the samples in relation to several not included concomitant maternal and neonatal risk factors, and the lack of severe ROP (stage 4 and 5) cases.

#### Glucose infusion in preterm infants

In newborns, especially if preterm [109], persistent altered blood glucose values (hypoglycemia and hyperglycemia [110] can be responsible for serious short and long-term consequences [111]. In preterm infants weighing < 2500 gr, the link between parenteral nutrition with three different glucose infusion rates 5- < 7, 7–13 and > 13–15 g/Kg/day, and selected neonatal morbidities and mortality, was studied [112]. Main results from univariate and multivariate logistic regression analyzes showed that glucose infusion of 5- < 7 g/kg/day in the first week, was an independent variable that significantly increased the risk of hypoglycemia (*P* = 0.010) and reduced the risk of sepsis (*P* = 0.026).

#### Respiratory distress syndrome

Term neonates can present neonatal respiratory distress syndrome (NRDS) with well described maternal and neonatal risk factors [113–115]. In this prospective case–control study, the authors compare 55 newborns with NRDS diagnosis and 79 non-NRDS as controls [116]. Main risk factors associated with the incidence and severity of NRDS were male gender (*p* = 0.045), elective caesarean section (*p* = 0.01) and early-onset infection (*p* = 0.055), while hypotension and pulmonary hypertension were associated with longer duration of parenteral nutrition and higher rate of blood transfusions. Main strength of this study is the prospective recruitment of participants and related data collection, while main limit is not have included information or correlation with severity predictive tools as Clinical Risk Index for Babies.

#### Delayed cord clamping

Delayed cord clamping (DCC) [117] is recommended for both preterm [118] and vigorous term neonates [119]. In a retrospective study [120] on 796 women and their term neonates delivered by cesarean section, the authors compare the effects of early versus DCC (< 30, 30–60 and 61–120 s), on short-term neonatal hematological status (hemoglobin and hematocrit) and jaundice (transcutaneous bilirubin levels on 0 to 5 days of life and the rate of phototherapy). The results showed that in the whole DCC cohort (30–120 s), clamping merely increased the transcutaneous bilirubin level of neonates on the day of birth and in the DCC at 30–60 is a higher neonatal hemoglobin level on day 3 and an increased rate of neonatal polycythemia, without a higher rate of phototherapy. The authors conclude that DCC at 30–60 s should be an optimal time in cesarean section, which could benefit the neonates in the long term.

#### Hypercalcemia and hypophosphatemia

Both hypercalcemia and hypophosphatemia could be deleterious in newborns [121, 122]. Improda et al. [123] describe a premature infant presenting with early severe hypercalcemia. In this patient, after a careful evaluation of the various pathogenetic hypotheses, according to the most compatible with laboratory data and the response to the therapeutic interventions, the authors ascribe to hypophosphatemia the key pathogenic factor [124]. The etiopathogenetic mechanism by which hypophosphatemia should have act was the inhibition of the secretion of FGF23, leading to increased activity of 1-alfa hydroxylase with production of 1,25(OH)2D, responsible for hypercalcemia. This article enriches the knowledge for the reader by providing useful and interesting insights into the correct monitoring and supplementation of phosphate and calcium in the first week of life in premature infants.

#### Necrotizing enterocolitis

Savarino et al. [125] described the clinical characteristics and the different therapeutic paths undertaken of 18 preterm infants with necrotizing enterocolitis. They reported age of onset, duration of previous enteral, as well as total parenteral feeding following surgery. In this population 77.8% received surgery and the overall survival rate was 55.5%. The absence of infants with stage I necrotizing enterocolitis may be related, to a failure of early diagnosis, being some of them out born, while the high prevalence of necrotizing enterocolitis at stage III (78%) to the presence of a Pediatric Surgery Ward in the same hospital. The discussion provides updates and considerations on enteral and parenteral feeding as well as medical and surgical treatment for necrotizing enterocolitis [126–128].

#### Hypertrophic cardiomyopathy

Hypertrophic cardiomyopathy accounts for 25 to 40% of all pediatric cardiomyopathy cases with the highest incidence in pediatric population in children < 1 year. The authors [129] take a cue from two newborns of diabetic mother suffering from hypertrophic cardiomyopathy to address the diagnostic and therapeutic approach. Hypertrophic cardiomyopathy may be characterized by recovery [130] or by progressive worsening [131, 132] that require skills in genetic and metabolic diagnosis along with a careful follow-up, as reported in the neonate with Pompe disease. An important message for the neonatologist is that early onset hypertrophic cardiomyopathy shows a high incidence of inborn errors of metabolism and neuromuscular disorders and that about 50% of hypertrophic cardiomyopathy cases under one year of age remains idiopathic.

#### Asymptomatic hypoglycemia

Asymptomatic hypoglycemia is a very dangerous condition, since it may cause, if prolonged, potential short and long-term adverse outcomes [133]. In a retrospective/prospective study on asymptomatic hypoglycemia in neonates > 35 weeks’ gestational age, Meneghin et al. [134] compared a previous standard approach [135] based on human or artificial milk and in selected cases on glucose infusion, and administration of 40% oral dextrose gel at 200 mg/kg followed by breast-feeding, squeezed human milk or artificial milk. Advantages of the latter approach were evident for both primary outcomes specifically occurrence of NICU admission (*p* = 0.001) and requirement of intravenous glucose (*p* = 0.01), as well as for the secondary outcome of exclusive breastfeeding at discharge (*p* = 0.02). The paper included clear and informative flow charts describing the two approaches that can be considered a valid starting point for a more adequate management of neonatal hypoglycemia [136].

#### Congenital hypothyroidism screening

Congenital hypothyroidism is an endocrine disease affecting 1:2000–1:3000 newborns in Italy. Early identification of congenital hypothyroidism [137] is based on the neonatal screening [138], and the choice of the TSH cutoff value is the discriminating element [139]. In this retrospective analysis of neonatal screening data, Maggio et al. [140] compared the number of congenital hypothyroidism diagnosis if the TSH cutoff was considered as ≥ 6—< 7 mU/L, ≥ 7—< 10 mU/L and ≥ 10 mU/L. Main conclusion of the study was that adopting a TSH cutoff ≥ 6 mU/L allowed recognizing 20.7% of neonates with confirmed high TSH levels, otherwise not recruited by the employed TSH cutoff > 7 mU/L. The authors detected additional cases of permanent congenital hypothyroidism, a number of which showed defects of thyroid embryogenesis and severe hypothyroidism at the confirmation of the diagnosis by serum levels of fT4, fT3 and TSH and by ultrasound and/or scintigraphy.

### Neurology. 1- Autism; 2-Plagiocephaly; 3- Post-infectious neurological syndromes; 4- Neuroblastoma; 5- Anorexia nervosa; 6- Epilepsy; 7- Fetal alchool spectrum disorder; 8- Cognitive functions

#### Autism

Autism spectrum disorder is a neurodevelopmental disorder displaying individual impairments in social interaction, communication skills, interest, and behaviours [141]. The autism can be diagnosed through The Autism Diagnostic Observation Schedule-Generic, a standardized assessment of social interaction, communication, play, and imaginative use of materials which considers the level of expressive language [142]. Most children who undergo the correct treatment at preschool age, after some months can greatly improve and do not show the initial behaviors, typical of the autism spectrum disorders. It has been increasingly recognized that it is important to evaluate the presence of positive predictive factors of outcome. Di Rienzo et al. [143] showed that the indicators were IQ, emotional contagion, understanding of others’ intentions and level of play achieved. To achieve a good outcome at least 3 predictive indicators must be present. Better verbal ability predicted better communication, socialization and daily living skills, global adaptive functioning, while better nonverbal ability predicted better motor skills. Autism spectrum disorder symptom severity has not proven to be a predictor of outcome [144]. Klinger et al. [145] found that treatment characteristics, including intensity and duration, are the most universally agreed upon predictors of treatment outcome, whereas an additional predictor of outcome which demonstrates congruent findings across studies is parental involvement. In conclusion, at preschool age, a therapeutic path for autism spectrum disorders must be considered fundamental for intervening in the affective-relational component, since at that age the brain has the potential to change structurally and functionally.

#### Plagiocephaly

Positional plagiocephaly, or non-synostotic plagiocephaly, is the most common cranial asymmetry. It results from mechanical factors applied, over a period in utero, at birth, or postnatally. It is often associated with excessive time in supine and with congenital muscular torticollis. Children with positional plagiocephaly are more likely to develop several conditions such as postural compensations, muscle flexibility and balance alterations, visual dysfunctions, temporomandibular dysfunctions, mandibular and occlusal asymmetries, neurodevelopmental alterations, lower cognitive and academic results, and language acquisition deficit. For this reason, it is important to start treatment early, when children are younger than 6.5 months [146]. Rogers et al. [147] showed that there was a restriction of active cervical rotation in all the cases of plagiocephaly studied, even without a previous diagnosis of congenital muscular torticollis, thus it is useful to use manual manouvres. The first line treatment recommended in the guidance reviewed for both positional plagiocephaly and congenital muscular torticollis include parent education and support, positioning/tummy-time, and physical therapy, followed by helmet therapy as a second line of treatment for infants with moderate to severe and persisting asymmetry. The Congress of Neurological Surgeons’ guidance recommended to use manual therapy and repositioning rather than positioning pillows [148]. A recent randomized controlled trial shows that adding manual therapy to a caregiver educational program is associated with a better outcome in terms of neck movement in positional plagiocephaly. The technique applied consists in letting the baby’s head rest on the hands of the practitioner. Both fourth and fifth fingers were positioned on the condylar area of the occipital bone, the middle finger on the articular processes of the axis, the index fingers on the articular processes of the cervical vertebrae below C2. The thumbs were placed on the anterior side of the transverse processes of the atlas to cause a very gentle dorsal positioning of the atlas, following the active and spontaneous movements of the baby [149].

#### Post-infectious neurological syndromes

Post-infectious neurological syndromes are heterogeneous neurological disorders with post or para-infectious (within 15 days from the infectious event) onset. These include the Acute Disseminated Encephalomyelitis, the Mild Encephalitis/Encephalopathy with Reversible Splenial Lesion, the Clinically Isolated Syndrome, and the Autoimmune Encephalitis and Necrotizing Encephalitis. These syndromes are characterized by demyelination, caused by immune-mediated reactions against cerebral, spinal cord and optic nerves white matter. The diagnosis may be difficult because there are no officially recognized guidelines. The only existing criteria have been published by the IPMSSG (reviewed in 2013) [150] and include pediatric multiple sclerosis and immune-mediated central nervous system demyelinating disorders. Tardieu et al. published a consensus definition for pediatric multiple sclerosis and other demyelinating disorders in childhood and propose to incorporate in IPMSSG criteria the 2010 McDonald criteria, when specific features are present [151]. Nevertheless, post-infectious neurological and multiple sclerosis show different clinical, neurobiological, and magnetic resonance imaging features [152]. No documents have ever mentioned specifically the post infectious neurological syndrome. Bozzola et al. [153] tried to provide with some indications and criteria for establishing a correct diagnosis. They suggested to consider the following parameters: neurological symptoms, timing of disease onset, blood and cerebral spinal fluid laboratory tests (positive for pathogenic IgM/IgG as a previous infection; positivity of autoantibodies; negative blood culture; negative cerebral spinal fluid culture, to exclude meningitis or acute infections), magnetic resonance imaging (which seems fundamental, even in the absence of clinical or laboratory diagnostic findings). Magnetic resonance imaging remains the most important criteria, even though other laboratory tests demonstrating the previous infection should be considered.

#### Neuroblastoma

Neuroblastoma is a heterogeneous solid tumor that arises in the sympathetic nervous system. Neuroblastoma tumors most commonly develop in the abdomen and are most frequently localized in the adrenal glands [154]. Clinical symptoms vary, depending on the site of the primary tumor and may include an abdominal mass, abdominal pain, respiratory distress, or neurological symptoms due to spinal cord involvement [155]. It has high heterogeneity, hidden onset, and poor prognosis [156]. A previous clinical study has demonstrated that 50% of children with metastatic neuroblastoma had chromosome abnormalities concerning chromosome 21, 10, or 11, with abnormalities on chromosome 10 being the most frequent [157]. Jang et al. [158] described the clinical characteristics of children with neuroblastoma and abnormal chromosome 10. The study showed that the overall survival rate of neuroblastoma children with abnormal chromosome 10 was significantly lower than that children with normal chromosome 10. The site 10q22 was linked to all structural abnormalities. This indicates that the site 10q22 may have tumor suppressor or oncogenic genes. Neuroblastoma patients with abnormal chromosome 10 were prone to orbital metastases. This study demonstrates that chromosome 10 can be used as a novel prognostic marker for neuroblastoma.

#### Anorexia nervosa

Over the past few decades, research has suggested a relationship between anorexia nervosa and autism spectrum disorder [159]. Autism spectrum disorder traits are present in approximately one third of cases with anorexia nervosa, and there is some evidence that autism spectrum disorder traits are associated with more severe eating disorder psychopathology [160]. Although anorexia nervosa and autism spectrum disorder refer to separate and diverse diagnostic categories, an underlying genetic vulnerability between the two disorders was proposed [161]. It is well established that certain neuropsychological functions are impaired in individuals with autism spectrum disorder (e.g., cognitive flexibility, such as set-shifting, and central coherence) and that those impairments are often recognised in the clinical presentation of anorexia nervosa [162]. Pruccoli et al. [163] have described the relationship between autism spectrum disorder traits, eating disorder psychopathology and the body mass index (BMI) in a population of young patients with anorexia nervosa at an Italian Regional Centre for Eating Disorders. Autism spectrum disorder traits and eating disorder psychopathology were investigated administering the Autism Diagnostic Observation Schedule-2, Autism Quotient and Eating Disorder Inventory-3. The results showed significant correlations between autism spectrum disorder trait and eating disorder psycopathology, that were independent of BMI, obsessive compulsive disorder and anti-psychotic treatments.

##### Epilepsy 

Eating epilepsy is a form of reflex epilepsy in which eating triggers seizures [164]. Eating epilepsy includes patients who had more than 50% of fits during or within 30 min of eating breakfast, lunch, or dinner [165]. It is a rare condition, with high prevalence in Sri Lanka [166]. The seizures can occur during any phase of the meal, and are likely provoked by olfactory, autonomic, or gustatory stimulus. As with other forms of reflex epilepsy the pathophysiology of eating epilepsy is unknown, but it is likely that the eating process triggers cortical activity leads to subsequent brainstem activation [167]; seizures usually arise in temporal limbic or perisylvian regions and may progress to secondary generalization [168]. Vercellino et al. [169] described two patients with symptomatic eating epilepsy, i.e., a 13-years-old boy with a bilateral perysilvian polymicrogyria and a 2-years-old boy with an underlying genetic disorder. In the latter case, a relevant contribution to the neurological phenotype is provided by de novo loss of function variants in CHD2, which can cause an early onset epileptic encephalopathy, hypotonia and dysmorphic features. The dysfunction of specific cortical regions in the contest of germline genetic alteration can lead to a hyperexcitation fostering the epileptogenesis.

##### Fetal alcohol spectrum disorders

Fetal alcohol spectrum disorders (FASD) are a group of conditions resulting from prenatal alcohol exposure. The clinical picture of FADS may vary, but the most affected brain functions include cognition [170, 171], especially executive functions [172] and social skills [173]. In this explorative study, Dylag et al. [174] aimed at investigating the sleep problems related to FASD. Forty patients were studied, through both Child’s Sleep Habits Questionnaire and polysomnography. The results show that a distorted sleep pattern characterised by sleep onset delay, night waking, parasomnias, sleep disordered breathing and daytime sleepiness, together with apneic/hypopneic events is often part of the clinical presentation of FADS. Further studies are needed to understand better the correlation between FADS and sleep problems.

#### Cognitive functions

Osika points up how the effects of technological devices and television on cognitive functions in several age groups [175], showing that spending time in front of a television may decrease cognitive skills such as language, concentration, but also emotional regulations [176, 177]. Prolonged usage of mobile phones can increase the risk of late language skills [178]. Furthermore, it is well known that screen usage also plays a major role in children's sleep [179, 180] affecting it both quantitatively (bedtime, sleep time, and total sleep time) and qualitatively (restful sleep or not) [181, 182]. The unrestrained usage of the devices has an important impact on essential parts of a child’s routine, such as learning time, sleep time, physical activity, and free play time. For all these reasons, it is fundamental to discourage screen exposure for very young children and advise parents on a more responsible use of technology.

### Nutrition. 1- Congenital chylothorax; 2- Formula feeding; 3- Macronutrients and micronutrients; 4- Breastfeeding technique

#### Congenital chylothorax

Congenital chylothorax is the accumulation of lymphatic fluid in pleural space. The management consists in respiratory support and dietary treatment. Dietary treatment provides the use of protein-based powdered formula with low long chain tryglicerides and high medium chain triglycerides, like monogen or basic fats. This takes away the possibility for the newborn (especially if premature) to benefit from breast milk. So, it was described in literature the possibility of skimming breast milk, to obtain a low-fat milk preserving its nutritional, immunologic, and psychosocial advantages [183]. A definitive guideline for medical management and therapeutic dietary intervention of congenital chylothorax is not available. A recent study has shown the outcome of six patients with chylothorax treated differently [184]. It showed positive effects of skimmed breast milk after resolution of pleural effusions. Three patients received skimmed breast milk: the first was associated with a favourable outcome, no recurrence of pleural effusion, adequate weight gain and a contented mother; the second was difficult to evaluate due to a diagnosed Noonan syndrome and the mother's subsequent decision to stop breastfeeding after two weeks; the third had a relapse of pleural effusion after the administration of skimmed milk. All patients started an additional treatment with somatostatin to speed up healing. Another positive effect is that in human milk somatostatin is found in high concentrations, reducing lymph production when breast feeding is continued in infants with chylothorax [185]. Then the positive effect on mother and child is proven, but a multicenter study in a larger number will be helpful and necessary.

#### Formula feeding

All major health organizations recommend breastfeeding as the optimal source of infant nutrition, with exclusive breastfeeding recommended for the first six months of life. After six months, complementary foods can be introduced. Most organizations recommend breastfeeding for at least one year, and the WHO recommends a minimum of 2 years, but less than 40% of infants below the 6 months are exclusively breastfeeding worldwide [186, 187]. Multiple investigations have demonstrated that breast feeding not only reduces the risk of death and disease in early life but has lasting health benefits through adult life [188, 189]. Taye et al. [189] assessed the Authors found that the educational status of mothers, timing of initiation breastfeeding, pre-lactal feeding, and delivery by cesarean section were significantly associated with formula feeding practice. Therefore, early initiation of breastfeeding, educating mothers about the risks associated with pre-lacteal feeding and supporting mothers who gave birth by cesarean section for exclusive breastfeeding should be encouraged at the community and institutional levels. Prevalence of formula feeding practice and its associated factors among mothers of an infant aged 0–6 months in Ethiopia.

##### Macronutrients and micronutrients

Macronutrients are mainly distinguished in carbohydrates, proteins, and lipids [190]. Micronutrients are components that do not provide a significant caloric intake and principally include vitamins and minerals [191]. In this interesting review, Savarino et al. [192] provided an up-to-date guide about the importance of both macro and micronutrients during paediatric age. During the first 6 months breast milk is the main source of energy, macro and micronutrients [193]. During weaning, the risk of an unbalanced diet increases, for this reason paediatricians should support the family during this process. Since preschool age is characterised by constant growth, the diet of the child should be checked by professionals that can ensure a varied diet with adequate portions. During puberty, a change in habits usually happens, with an increased risk for “bad habits”, like choosing “junk food” during meals “away from home”. In conclusion, each period of the paediatric age has specific requirements and characteristics, and clinicians should be able to ensure adequate support to families for children's nutrition.

#### Breastfeeding technique

Breastfeeding technique is explained as the mother’s and baby’s positioning, baby’s attachment to the breast, and suckling during breastfeeding, which are very important for effective breastfeeding [194, 195]. Ineffective breastfeeding technique is the leading cause of various problems related to breastfeeding and significantly affects both maternal and infants’ health [196]. Younger and primiparous mothers usually perform poorly effective breastfeeding. But women having counseling during antenatal care follow-up and immediately after delivery and not having breast problems applied breastfeeding effectively. In their observational study, Safayi et al. [197] showed that counseling had a significant contribution to effective breastfeeding. Moreover, it revealed that breastfeeding technique is deficient in women giving birth for the first time. In conclusion, authors recommend giving particular emphasis especially to younger mothers and primipara mothers to encourage adequate breastfeeding technique.

### Palliative care. 1- Medical complexity; 2- Cannabis

#### Medical complexity

Children with medical complexity are a population in need of pediatric palliative care. They have multiple chronic health problems that affect multiple organs, and they have high risk of adverse outcomes, both in the psychological and medical fields. A recent retrospective cross-sectional survey analysed prevalence and needs of children with medical complexity from 14 local health authorities of Emilia Romagna [198]. According to this survey, 51% of the children had neurological conditions, the single most frequent diagnosis was cerebral palsy. This prevalence was found also in a recent review on pediatric palliative care [199]. However, it is more common and faster to start palliative care to a child with cancer [200]. Children with medical complexity often need assistance as they have higher access to health services and assistance costs. Another data that emerges from the study is that there should be more integration between hospital and home care. The results in this literature review include home-based care and community-based services for children, which make it possible for children in need of palliative care to stay at home [201]. In addition, it is important to selecting patients in need in the most appropriate way possible and to give more attention to patient's family because the goal in both general and specific paediatric palliative care is to improve quality of life for both the child and his or her family [198].

#### Cannabis

Cannabis and cannabinoids preparations exert numerous therapeutic effects. They have antispastic, analgesic, antiemetic, neuroprotective, and anti-inflammatory actions, and are effective against certain psychiatric diseases [202]. Overall, trials demonstrated that cannabis significantly reduced the frequency of seizures compared to placebo [203]. Children and young adults with cancer reported using cannabis products for nausea and vomiting, depressed mood, sleep disturbances, pain, poor appetite, and weight loss [204]. Divisic et al. described a clinical experience of six patients in a pediatric palliative care who received a titrated plant extract of cannabis sativa for 1 year [205]. All patients received cannabis therapy for treatment-resistant epilepsy and chronic pain. In this experience, only mild and transient adverse event occurred: drownsiness, euphoria, restlessness, and tachycardia: the resolution of symptoms were either spontaneous or obtained by modifying the administration schedule. The experiences suggest that a titrated paint extract preparation of medical cannabis may be useful to control treatment-resistant paint and epilepsy in pediatric palliative care patients.

### Respiratory tract illnesses. 1- Cystic fibrosis; 2- Recurrent respiratory infections; 3- Respiratory syncytial virus; 4- Inhaled corticosteroids; 5- Foreign body aspiration; 6- Severe pneumonia

#### Cystic fibrosis

Cystic fibrosis (CF) is a multi-organ disease, which mainly affects the respiratory and digestive systems. It is a genetic disorder due to an altered gene, i.e., the CFTR gene. The sweat test is the gold standard for the diagnosis of CF, also used in newborn screening (NBS) programs and to assess the response to treatment with new modulators. Nevertheless, it is well known that sweat test may give false negative or positive results, with a rate ranging from 10 to 15% [206]. Patients with other underlying disorders, i.e., celiac disease, Klinefelter Syndrome, coprostasis in treatment with polyethylene glycol may show false positive sweat test. Cimbalo et al. have tried to explain the reason of this [207], discriminating false positive and false negative sweat test results into 3 categories, i.e., ‘‘likely’’, ‘‘probable but needs validation,’’ and ‘‘unlikely’’ results based on the number of cases reported and use of genetic testing to exclude CF. They concluded that clinicians must aware the possibility of other conditions, such as adrenal insufficiency, even if two CFTR mutations are identified [208]. They also recommend repeating the sweat test and/or genetic analyses twice, possibly in specialized laboratories [209]. The mutated protein produced by CFTR gene does not work properly and leads to the production of thick mucus and sweat very rich in salts. The secretions of people with CF contain purulent material, polymerized DNA, and filamentous actin (F-actin) proteins derived from dead inflammatory cells and epithelial cells and very little mucin. This has important therapeutic implications. Terlizzi et al. [210] have emphasized that at present symptomatic mucolytic treatment of patients with CF includes the inhalation of DNase, hypertonic saline or mannitol combined with chest physiotherapy. Several articles have shown that there is no superiority of hypertonic saline than other mucolytic agents, even if the benefits are that it’s an inexpensive, safe, and effective additional therapy [211, 212]. It is possible the use of more than one inhaled solution at the same time, to achieve more benefit since these agents have different mechanisms of action [213]. Accordingly, authors recommend the dornase alfa as the first choice in routine treatment and if the clinical response is inadequate, hypertonic saline is proposed alone or in combination with dornase alfa.

#### Recurrent respiratory infections

Paediatric respiratory tract infections are one of the most common reasons for physician visits and hospitalisation, and they are associated with significant morbidity and mortality. In most cases, infections occur with mild clinical manifestations and gradually improve by the age of 12 years old [214]. The diagnosis of recurrent respiratory infections (RRIs) is basically a diagnosis of exclusion of other chronic conditions, such as genetic pathologies, cystic fibrosis, congenital immunodeficiencies, malformations. Chiappini et al. [215] have promoted a consensus document with the aim to propose an updated definition and provide recommendations with the intent of guiding the physician in the complex process of diagnosis, management, and prevention of RRIs. Main findings are: 1) the use of oral probiotic formulations should not be recommended for the prevention of RRIs; 2) the routine vitamin C supplementation should not be used in the prevention of RRIs; 3) evidence available to date does not allow recommendation of the routine use of bacterial lysates for the prevention of RRIs; 4) although there few publications on the effects of influenza and anti-pneumococcal vaccinations specifically for the prevention of RRIs, they are strongly recommended; 5) nasal therapy with hyaluronic acid, thermal waters for the prevention of RRIs should not be discouraged; 6) the adeno-tonsillectomy is not recommended for the reduction of RRIs; 7) no studies are available on the efficacy of antibiotic prophylaxis in preventing RRIs.

#### Respiratory syncytial virus

Respiratory syncytial virus (RSV) is the most common respiratory agent in infants and young children worldwide. It is by far the most frequent cause of hospitalization in children younger than 2 years of age, particularly in those born prematurely or who have chronic lung disease or congenital heart disease [216]. RSV can cause severe lower respiratory tract infections, such as pneumonia and bronchiolitis, and it is associated with an increased risk of developing asthma and recurrent wheezing [217]. Azzari et al. [218] have reviewed the global RSV epidemiological data and current prevention strategies. Prevention strategies include maternal vaccines which can protect neonates from birth and during the RSV epidemic season. A prevention strategy based on passive immunoprophylaxis provide immediate protection of the child and last up to 5 months. Pediatric vaccines can be administered to all children, provide longer-term protection and limit RSV circulation within the pediatric population. The possible cooperation between maternal vaccination or passive immunoprophylxis to protect neonates from birth and subsequent pediatric vaccination to achieve more durable protection and limit RSV circulation.

#### Inhaled corticosteroids

A consensus document for the use of inhaled corticosteroids [219] was provided by experts from eight scientific societies, he Italian Society for Paediatrics (SIP), the Italian Society of Paediatric Respiratory Diseases (SIMRI), the Italian Society for Paediatric Allergy and Immunology (SIAIP), the Italian Society for Preventive and Social Paediatrics (SIPPS), the Italian Society of Paediatric Primary Care (SICuPP), the Italian Society of Adolescent Medicine (SIMA), the Italian Society of Paediatric Emergency Medicine (SIMEUP) and the Italian Federation of Paediatricians (FIMP). Through clinical questions and systematic review of the literature, the expert panel provided recommendations on the use of inhaled corticosteroids, in preschool wheezing, asthma [220], allergic and non-allergic rhinitis [221], acute and chronic rhinosinusitis [222], adenoid hypertrophy, laryngospasm. The panel recommended against of inhaled corticosteroids administration in acute infectious laryngitis, otitis media with effusion. This article is useful guidance for paediatricians and general practitioners.

##### Foreign body aspiration 

Foreign body aspiration (FBA) is a common and possibly life-threatening event [223], that could lead to serious clinical events such as pneumonia, bronchiectasis, lung abscess, atelectasis, or even death. The severity of the complications may be due to missed or delayed diagnosis and management [224]. In this retrospective cohort study, Fasseeh et al. [225] have proposed new criteria to predict FBA in children, together with the elaboration of a clinical algorithm that may help in decision making about the need and type of bronchoscopy in children presenting with potential FBA.

#### Severe pneumonia

Pediatric community acquired pneumonia (CAP) is frequently encountered by medical providers and is one of the most common reasons for hospital [226] and the major cause of pediatric mortality [227]. CAP is a pulmonary infectious disease acquired outside of the hospital, with viruses and bacteria as the most common pathogens [228]. Previous studies were focused on the identification of risk factors associated with severe CAP and thus facilitated the risk stratification of CAP patients [229]. Chen et al. [230] have investigated the age-specific risk factors for severe disease among children hospitalized with CAP. Birth history, including premature birth and low birth weight (< 2.5 kg), is a significant predictor of severe CAP. The breastfeeding acting is a protective factor for CAP, whereas formula feeding was associated with increased risk. The infants born within 6 months demonstrated a significantly higher risk of developing severe CAP than older children. The risk factors predicting disease severity among children hospitalized with CAP vary with age. An age-specific model should be developed for risk stratification of pediatric CAP patients, which could better guide the practice of precision medicine.

### Telemedicine. 1- Paediatric influencers

#### Paediatric influencers

In this study, Bozzola et al. [231] have evaluated the role of paediatric influencers in communicating information about children’s and adolescents' health [232]. The authors' study shows the potential role of influencers: following specific guidelines and criteria spreading health messages via paediatric influencers seems to be a successful strategy to support correct communication about children's and adolescents' health [233, 234]. In conclusion, any medical doctor and health care professional could promote health messages among patients and families, to achieve two significant results in one shot: spreading correct information and contrasting the spreading of fake news on sensible health topics.

## Conclusions

We have offered major advancements in the growing field of Pediatrics through the eyes of the Italian Journal of Pediatrics. The past year's papers have provided clear and incisive information on how can change children care. Impactful views and comprehensions will enhance the quality of care for childhood diseases.

## Data Availability

Data sharing is not applicable to this article as no datasets were generated or analysed during the current study.

## Abbreviations

CAP: Community acquired pneumonia
CF: Cystic fibrosis
DCC: Delayed cord clamping
EBV: Epstein-Barr virus
FBA: Foreign body aspiration
HLH: Hemophagocytic syndrome
LAIV: Live attenuated influenza vaccine
NICU: Neonatal intensive care units
NRDS: Neonatal respiratory distress syndrome
RRI: Recurrent respiratory infections
ROP: Retinopathy of prematurity
RSV: Respiratory syncytial virus
WHO: World Health Organization
